# Integrated analysis of 8-week glecaprevir/pibrentasvir in Japanese and overseas patients without cirrhosis and with hepatitis C virus genotype 1 or 2 infection

**DOI:** 10.1007/s00535-019-01569-7

**Published:** 2019-03-13

**Authors:** Atsushi Naganuma, Kazuaki Chayama, Kazuo Notsumata, Edward Gane, Graham R. Foster, David Wyles, Paul Kwo, Eric Crown, Abhi Bhagat, Federico J. Mensa, Tetsuya Otani, Lois Larsen, Margaret Burroughs, Hiromitsu Kumada

**Affiliations:** 1 Department of Gastroenterology, Takasaki General Medical Center, National Hospital Organization, 36 Takamatsu-cho, Takasaki-shi, Gunma 370-0829 Japan; 2 Hiroshima University Hospital, Hiroshima, Japan; 3 Fukuiken Saiseikai Hospital, Fukui, Japan; 4 Liver Unit, Auckland City Hospital, Auckland, New Zealand; 5 Queen Mary University of London, Barts Health, London, UK; 6 Denver Health Division of Infectious Diseases, University of Colorado, Denver, CO USA; 7 Division of Gastroenterology and Hepatology, Stanford University, Palo Alto, CA USA; 8 AbbVie Inc., North Chicago, IL USA; 9 Toranomon Hospital, Tokyo, Japan

**Keywords:** Chronic hepatitis C, Antiviral agents, Protease inhibitors, Comorbidity, Sustained virologic response

## Abstract

**Background:**

Chronic hepatitis C virus (HCV) infection with genotypes (GT) 1 and 2 accounts for over 50% of HCV infections globally, including over 97% of all HCV infections in Japan. Here, we report an integrated analysis of efficacy and safety of 8-week treatment with the all-oral, fixed-dose combination of the direct acting antivirals (DAA), glecaprevir and pibrentasvir (G/P), in DAA-naïve Japanese and overseas patients without cirrhosis and with HCV GT1 or GT2 infection.

**Methods:**

Data from 899 DAA-naïve patients without cirrhosis and with HCV GT1 or GT2 infection treated with G/P (300/120 mg) for 8 weeks in the six Phase 2 or 3 overseas or Japan-only clinical trials were included. All patients who received ≥ 1 dose of G/P were included in an intent-to-treat (ITT) analysis. The objectives were to evaluate rate of sustained virologic response 12 weeks post-treatment (SVR12) and safety of the 8-week regimen in the ITT population.

**Results:**

Overall, SVR12 was achieved by 98.9% (889/899) of DAA-naïve patients without cirrhosis, including 99.2% (597/602) of GT1-infected and 98.3% (292/297) of GT2-infected patients. Less than 1% (2/899) of patients overall and no Japanese patients experienced virologic failure. SVR12 rate was > 97% for patients regardless of baseline characteristics, and common comorbidities or co-medications. Overall, < 1% (2/899) discontinued G/P due to an adverse event (AE) and 1.6% (14/899) of patients experienced a serious AE.

**Conclusions:**

8-week G/P treatment is safe and efficacious in DAA-naive patients without cirrhosis and with HCV GT1 or GT2 infection, demonstrating high SVR12 rates regardless of baseline patient and disease characteristics.

**ClinicalTrials.gov identifiers:**

The trials discussed in this paper were registered with ClinicalTrials.gov as follows: NCT02707952 (CERTAIN-1), NCT02723084 (CERTAIN-2), NCT02243280 (SURVEYOR-I), NCT02243293 (SURVEYOR-II), NCT02604017 (ENDURANCE-1), NCT02738138 (EXPEDITION-2).

**Electronic supplementary material:**

The online version of this article (10.1007/s00535-019-01569-7) contains supplementary material, which is available to authorized users.

## Introduction

Chronic hepatitis C virus (HCV) infection affects 71 million individuals worldwide including approximately 1.5 million in Japan [1, 2]. Genotypes (GT) 1 and 2 comprise approximately 55% of all HCV infection globally, including > 97% of all HCV infections in Japan [3, 4]. Patients with chronic HCV infection commonly have comorbidities and use co-medications, particularly elderly patients who constitute a large portion of the HCV-infected patient population in Japan [5]. Untreated patients with chronic HCV infection are at risk for fibrosis progression, cirrhosis, and hepatocellular carcinoma (HCC), especially elderly patients who have a greater prevalence of advanced fibrosis [6–8]. Achievement of sustained virologic response (SVR) has been shown to improve liver function and decrease risk of liver-related and all-cause mortality [9, 10].

Despite the advent of safe and effective direct-acting antivirals (DAA) for treating chronic HCV infection, there was still a need for simplified treatment regimens suitable for shorter treatment durations in patients regardless of baseline viral or patient characteristics. Prior to 2017, treatment guidelines in the United States of America, Europe, and Japan recommended treatment durations of 12 weeks or more for most approved HCV regimens in DAA-naïve patients without cirrhosis [11–13]. Shorter treatment durations could improve patient adherence since, amongst patients with HCV, adherence has been shown to decrease over the course of 12-week treatment [14]. Both previous and current guidelines also recommend assessing for comorbidities and co-medication use prior to treatment initiation due to the potential for drug–drug interactions that may impact treatment response [11–13, 15–17]. Lastly, prior to 2017, no pangenotypic DAA regimen was approved in Japan. Thus, there was an unmet need, especially in Japan, for a shorter duration and pangenotypic regimen that is highly efficacious regardless of baseline viral or patient characteristics.

The DAA combination of glecaprevir (NS3/4A protease inhibitor developed by AbbVie and Enanta) and pibrentasvir (NS5A inhibitor), co-formulated as the once-daily, pangenotypic regimen of glecaprevir/pibrentasvir (G/P), was recently approved for patients with chronic HCV GT1-6 infection including those with compensated cirrhosis, chronic kidney disease (CKD), and prior DAA failure in Japan and overseas. In vitro, both glecaprevir and pibrentasvir exhibit a high barrier to resistance, since they retain their nanomolar and picomolar potencies, respectively, against resistance-associated substitutions (RASs) including the NS5A RASs Y93H and L31M/V [18–20]. In its overseas clinical trials conducted outside of Japan, 8- and 12-week treatment in patients without cirrhosis and with HCV genotypes 1–6 achieved similarly high (≥ 95%) SVR12 rates [21]. Integrated analyses across these patients also confirmed that there was no significant difference in SVR12 rates between the 8- and 12-week durations for any of the baseline characteristics analyzed including fibrosis stage and the presence of baseline polymorphisms in NS3 or NS5A [21]. In 2 separate active-controlled clinical trials conducted in Japan, 8-week G/P treatment demonstrated non-inferiority to previous standards of care for DAA-naïve Japanese patients without cirrhosis and with GT1 or GT2 infection [22, 23], leading to the recommendation for 8-week treatment in these patients regardless of baseline viral or patient characteristics [17].

Here, we present an integrated analysis of the six overseas and Japan-only Phase 2/3 clinical trials that evaluated 8-week G/P treatment in order to summarize the safety and efficacy of 8-week G/P treatment and to assess its efficacy by baseline patient and disease characteristics.

## Methods

### Analysis set

This is an integrated analysis of data from the six Phase 2 and 3 clinical trials that assessed the efficacy and safety of 8 weeks of glecaprevir and pibrentasvir in treatment-naïve or pegIFN/RBV-experienced patients without cirrhosis and with HCV GT1 or GT2 infection. Patients received glecaprevir 300 mg and pibrentasvir 120 mg co-administered (Phase 2) or co-formulated G/P (300 mg/120 mg; Phase 3) dosed orally as a three-pill, once-daily regimen taken with food for 8 weeks. All patients provided written informed consent. Studies were designed and conducted in accordance with the Good Clinical Practice guidelines, Declaration of Helsinki, and applicable local regulation, with approval from independent ethics committees or institutional review boards at all study sites. The detailed methodology and primary outcomes have been published previously for all studies (CERTAIN-1 [NCT02707952], CERTAIN-2 [NCT02723084], SURVEYOR-I [NCT02243280], SURVEYOR-II [NCT02243293], ENDURANCE-1 [NCT02604017], and EXPEDITION-2 [NCT02738138]) [22–27]. All authors had access to data from this integrated analysis, and reviewed and approved the final manuscript for submission.

### Patients

Patients with GT1 or GT2 infection were at least 18 years of age and positive for anti-HCV antibody with a plasma HCV RNA viral load ≥ 10000 IU/mL in Phase 2 or ≥ 1000 IU/mL in Phase 3 at the Screening Visit. Eligibility for 8-week treatment required that patients were without cirrhosis, and were either HCV treatment-naïve or had prior treatment experience with interferon (IFN)/pegIFN ± ribavirin (RBV). The absence of cirrhosis was defined using a hierarchal approach in all studies, where sites utilized liver biopsy, transient elastography, or screening Fibrotest and aspartate aminotransferase (AST)–platelet ratio index (APRI) as outlined in the Supporting Information. In CERTAIN-1 and -2 only, the absence of cirrhosis could also be confirmed by a Screening Discriminant Score (*z*) < 0 as outlined in the Supporting Information.

### Assessments

HCV genotype was determined using the Versant^®^ HCV Genotype Inno LiPA Assay, Version 2.0 or higher (LiPA; Siemens Healthcare Diagnostics, Tarrytown, NY), and confirmed by phylogenetic analysis of viral sequences. Baseline viral load and SVR12 were assessed using PCR to quantify plasma HCV RNA; assay details are described in the Supporting Information. Patients with key baseline substitutions were classified as anyone with a resistance-associated variant in NS3 (at amino acid positions 155, 156, and 168) or NS5A (at amino acid positions 24 (GT2 only), 28, 30, 31 (GT1 only), 92 (GT2 only), and 93).

Safety was evaluated through physical examinations, laboratory testing, and monitoring of adverse events. Non-serious and serious AEs were monitored throughout G/P treatment until 30 days post-treatment and up to 24 weeks post-treatment, respectively. Treatment-emergent adverse events (TEAEs) were defined as any AE with an onset date after the first G/P dose and no more than 30 days after the last G/P dose. All AEs were coded using the Medical Dictionary for Regulatory Activities (MedDRA) version 20.0 and were assessed for their relationship to G/P by study investigators.

### Endpoints

The primary endpoint was the rate of sustained virologic response (HCV RNA < lower limit of quantification) at 12 weeks post-treatment (SVR12) for both Japanese and overseas patients by genotype. Secondary endpoints included the number of on-treatment virologic failures and relapses. Subgroup analyses further evaluated overall SVR12 rates by baseline patient and disease characteristics including presence of advanced fibrosis, comorbidities, and co-medication use. The presence of advanced fibrosis was assessed using the FIB-4 index that is a validated, non-invasive method with high specificity (> 97%) for identifying patients with advanced fibrosis (F3/F4) using > 3.25 as a cut-off and high negative predictive value (> 90%) for excluding advanced fibrosis using a cut-off of < 1.45 compared to liver biopsy [28, 29]. Safety was evaluated in both Japanese and overseas patients by assessing the number and percentage of patients with treatment-emergent adverse events and laboratory abnormalities.

### Statistical analyses

In this retrospective analysis of data from phase 2 and 3 clinical trials, efficacy and safety analyses were performed for the intention-to-treat (ITT) population that included all patients assigned to 8-week G/P (300/120 mg) treatment who received ≥ 1 dose of G/P. The number and percentage of patients in this ITT population achieving SVR12 for each genotype and overall were summarized with two-sided 95% confidence intervals calculated using the Wilson score method.

## Results

### Baseline patient demographics and characteristics

This analysis included 899 patients with chronic hepatitis C genotypes 1–2 from six Phase 2 and 3 clinical trials who were treated with G/P for 8 weeks. Among these 899 patients, 602 (67%) and 297 (33%) patients were chronically infected with HCV genotype 1 and 2, respectively. Most patients (n, %) were either white (555, 62%) or Asian (287, 31%), treatment-naïve (673, 75%), and < 65 years of age (725, 81%); the median FIB-4 index was 1.4. The analysis population included 69 (8%) patients with advanced fibrosis as defined by FIB-4 > 3.25 as well as 295 (33%) patients with cardiovascular disease and 90 (10%) taking proton pump inhibitors. Table 1 describes full patient characteristics both overall and by HCV genotype.

**Table 1:** Baseline demographics and disease characteristics

Characteristic	GT1 *N* = 602	GT2 *N* = 297	Overall *N* = 899
Male, *n* (%)	304 (50)	141 (47)	445 (49)
Race, *n* (%)			
White	384 (64)	171 (59)	555 (62)
Black or African American	33 (5)	13 (4)	46 (5)
Asian^a^	180 (30)	107 (36)	287 (31)
Other	5 (< 1)	6 (2)	11 (1)
Age, median (range), years	54 (19–86)	57 (21–83)	55 (19–86)
Age distribution, *n* (%)			
≥ 65	113 (19)	61 (21)	174 (19)
≥ 75	31 (5)	13 (4)	44 (5)
BMI, median (range), kg/m^2^	24.7 (16.2–41.4)	25.3 (14.2–65.7)	24.8 (14.2–65.7)
HCV treatment history			
Treatment-naïve	411 (68)	262 (88)	673 (75)
Treatment-experienced^b^	191 (32)	35 (12)	226 (25)
Baseline HCV RNA level, median (range), log_10_ IU/mL	6.2 (1.2–7.6)	6.6 (0.7–7.6)	6.3 (0.7–7.6)
FIB-4 index, median (range)	1.4 (0.3–7.8)	1.5 (0.3–7.9)	1.4 (0.3–7.9)
FIB-4 index			
< 1.45	317 (53)	147 (49)	464 (52)
1.45–3.25	244 (41)	122 (41)	366 (41)
> 3.25	41 (7)	28 (9)	69 (8)
IL28B			
CC	217 (36)	165 (56)	382 (42)
Non-CC	385 (64)	132 (44)	517 (58)
Presence of key baseline substitutions, *n* (%)			
NS3 only^c^	9 (2)	2 (< 1)	11 (1)
NS5A only^d^	81 (14)	26 (9)	107 (13)
NS3 + NS5A^c,d^	1 (< 1)	1 (< 1)	2 (< 1)
Baseline NS5A Y93H, *n* (%)	54 (9)	0	54 (6)
History of disorders, *n* (%)			
Hypertension	153 (25)	42 (14)	195 (22)
Gastroesophageal reflux disease	45 (7)	7 (2)	52 (6)
Hyperlipidemia	13 (2)	14 (5)	27 (3)
Diabetes	41 (7)	20 (7)	61 (7)
Cardiovascular disease	187 (31)	108 (36)	295 (33)
Chronic kidney disease stage 4 or 5^e^	3 (< 1)	7 (2)	10 (1)
Concomitant medications, *n* (%)			
Calcium channel blockers	77 (13)	46 (15)	123 (14)
Angiotensin II receptor blockers	67 (11)	23 (8)	90 (10)
Statins	32 (5)	17 (6)	49 (5)
Proton pump inhibitors	53 (9)	37 (12)	90 (10)

*BMI* body-mass index, *HCV* hepatitis C virus, *FIB-4* fibrosis-4

^a^Includes 132 GT1-infected and 104 GT2-infected Japanese patients from CERTAIN-1 and CERTAIN-2 Phase 3 clinical trials

^b^Prior treatment experience with interferon (IFN)/pegIFN ± ribavirin (RBV)

^c^Defined as having any baseline NS3 resistance-associated variant (at amino acid positions 155, 156, and 168) at ≥ 15% NGS detection threshold

^d^Defined as having any baseline NS5A resistance-associated variant (at amino acid positions 24 (GT2 only), 28, 30, 31 (GT1 only), 92 (GT2 only), and 93) at ≥ 15% NGS detection threshold

^e^Defined as estimated glomerular filtration rate (eGFR) < 30 mL/min/1.73 m^2^ at screening

### Efficacy outcomes

**Fig. 1:**
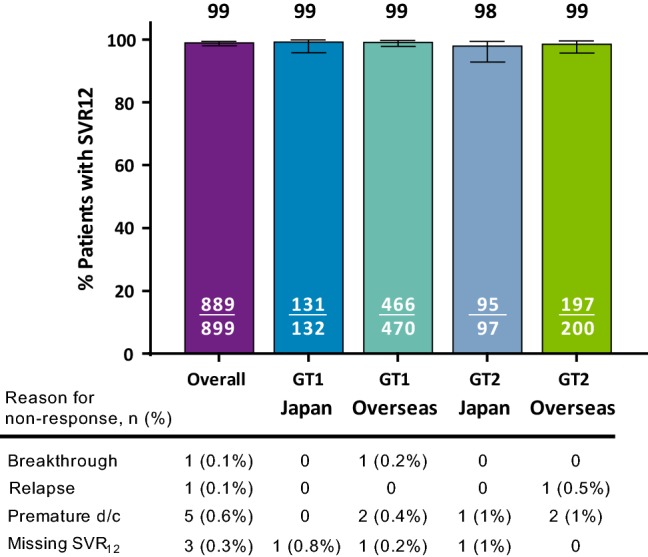
*Overall SVR12 comparing Japan and overseas rates by genotype.* Efficacy of 8-week G/P treatment defined as SVR12 is reported for both Japan and overseas patients by genotype using an ITT analysis. The table lists the reason for non-response including virologic (breakthrough or relapse) and non-virologic failure (premature discontinuation or missing SVR12) for each group. Premature d/c, Premature discontinuation

Overall, the rate of SVR12 by ITT analysis was 98.9% (889/899; 95% CI = 98.0–99.4) with numerically comparable SVR12 rates between Japan and overseas patients with HCV GT1 (Japan: 99.2%, 131/132, 95% CI = 95.8–99.9; overseas: 99.1%, 466/470, 95% CI = 97.8–99.7) and GT2 infection (Japan: 97.9%, 95/97, 95% CI = 92.8–99.4; overseas: 98.5%, 197/200, 95% CI = 95.7–99.5) (Fig. 1). Of the 10 patients who did not achieve SVR12, no Japanese patients and only 2 (< 1%) patients overall experienced virologic failure, including one on-treatment virologic failure and one relapse. One overseas patient with GT1a infection and prior pegIFN/RBV treatment experienced on-treatment virologic failure by treatment day 49, leading to premature discontinuation of G/P. One overseas patient with GT2a infection and prior pegIFN/RBV treatment had relapse by post-treatment week 12. More information on the virologic failures is included in Table S1.

**Fig. 2:**
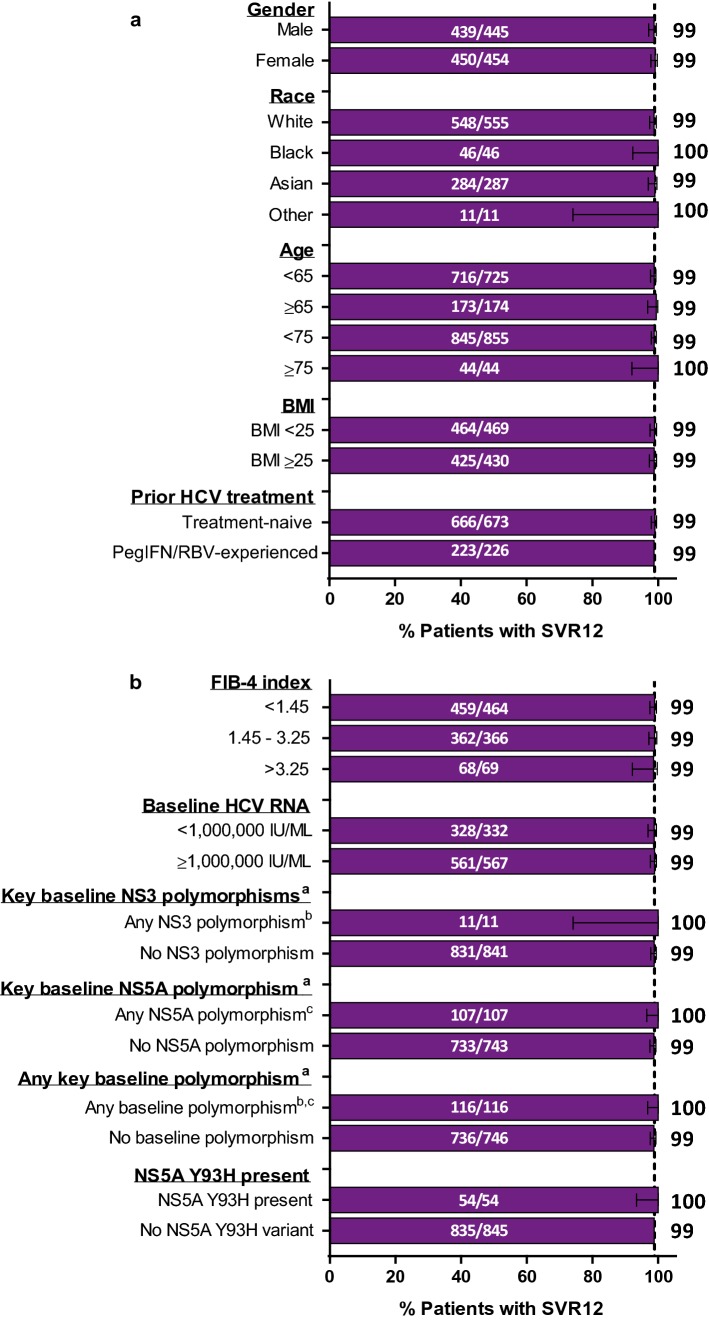
*SVR12 by baseline patient and disease characteristics.* Efficacy of 8-week G/P treatment reported by baseline patient (**a**) and disease (**b**) characteristics using an ITT analysis. The dashed line represents the overall SVR12 for 899 patients included in the analysis. ^a^All patients with missing data for baseline polymorphisms achieved SVR12. ^b^Defined as having any baseline NS3 resistance-associated variant (at amino acid positions 155, 156, and 168) at ≥ 15% NGS detection threshold. ^c^Defined as having any baseline NS5A resistance-associated variant (at amino acid positions 24, 28, 30, 92, and 93) at ≥ 15% NGS detection threshold

Rates of SVR12 by ITT analysis were > 98% regardless of baseline variables including gender, race, age, body-mass index (BMI), prior HCV treatment history, FIB-4 index, and viral load (Fig. 2). All patients with baseline NS3 or NS5A polymorphisms in key resistance-associated variants including 54 patients with the NS5A Y93H RAS achieved SVR12. Notably, SVR12 was achieved at numerically comparable rates between patients with more advanced fibrosis (FIB-4 > 3.25; 98.6%) and those without advanced fibrosis (FIB-4 < 1.45; 98.9%).

**Fig. 3:**
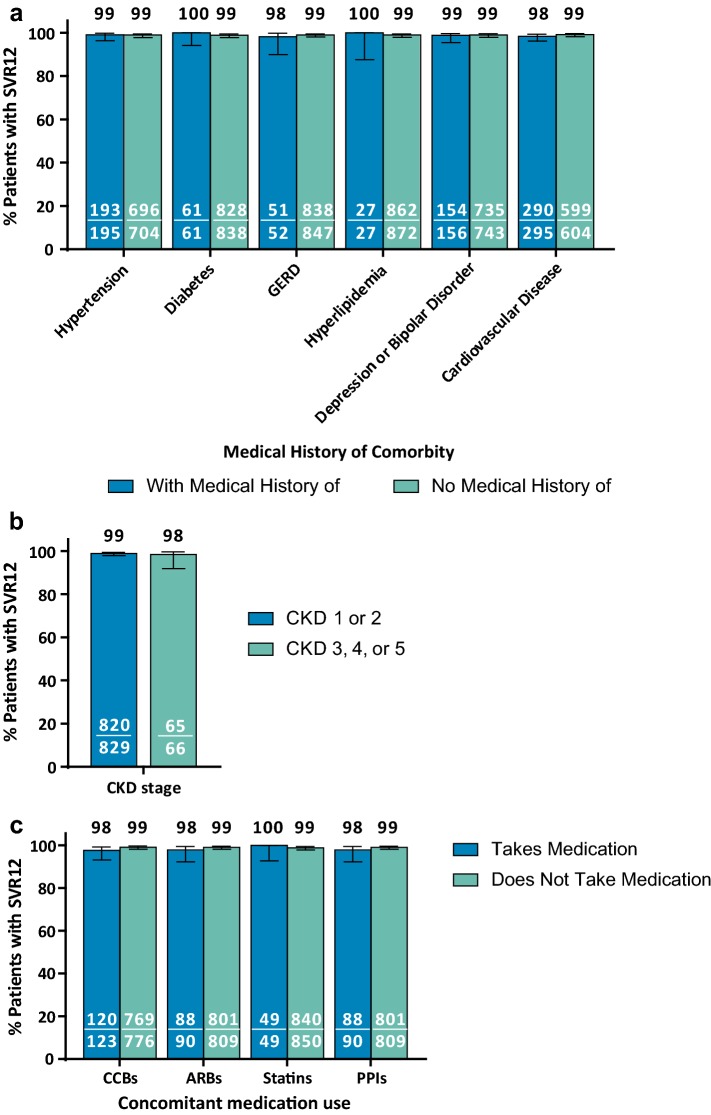
*SVR12 by comorbidities and co*-*medications.* Efficacy of 8-week G/P treatment, defined as SVR12, is reported by comorbidity (**a**–**b**) and co-medications (**c**) using an ITT analysis. GERD, gastroesophageal reflux disease; CKD, chronic kidney disease; *CCB* calcium channel blockers, *ARB* angiotensin receptor blockers, *PPI* proton-pump inhibitors

SVR12 rates by ITT analysis remained > 97% for patients with common comorbidities and co-medications (Fig. 3). SVR12 rates were numerically comparable for patients with or without the corresponding comorbidities (Fig. 3a), CKD stages 1/2 or 3/4/5 (Fig. 3b), and concomitant medications (Fig. 3c), respectively. Of note, all 10 Japanese patients with chronic kidney disease (CKD) stage 4 or 5 achieved SVR12.

### Safety outcomes

Overall, 541 of 899 (60%) patients reported ≥ 1 treatment-emergent adverse event (AE) including 125 of 229 (55%) Japanese patients and 416 of 670 (62%) patients from overseas (Table 2), of which most Japanese (94%; 118/125) and most overseas (96%; 400/416) patients experienced an AE with a maximum severity of mild (Grade 1) or moderate (Grade 2). Overall, the most common AEs occurring in ≥ 5% of patients were headache (12%), fatigue (9%), viral upper respiratory tract infection (7%), and nausea (7%). Headache and fatigue appeared to be reported less frequently in Japanese patients than in those from overseas (6% compared to 15% for headache and < 1% compared to 12% for fatigue); however, there was a higher rate of viral upper respiratory tract infections in Japanese patients compared to those from overseas (12% compared with 5%, respectively). Serious AEs were reported in 14 (2%) patients occurring at similar rates between Japanese (1%) and overseas (2%) patients; none were considered related to G/P by the study investigator. Two (< 1%) patients prematurely discontinued G/P due to AEs. One patient from overseas discontinued study drug due to a serious AE of adenocarcinoma on treatment day 29 and subsequently died 60 days post-treatment as a result of this serious AE; this was considered not related to G/P. A Japanese patient experienced non-serious AEs of nausea and vomiting related to G/P that led to treatment discontinuation on day 18.

**Table 2:** Adverse events in Japanese and overseas patients

Event	Japan GT1-2 *N* = 229	Overseas GT1-2 *N* = 670	Overall *N* = 899
Any AE, *n* (%)	125 (55)	416 (62)	541 (60)
Any DAA-related^a^ AE	50 (22)	234 (35)	284 (32)
Any serious AE	3 (1)	11 (2)	14 (2)
Any DAA-related^a^ serious AEs	0	0	0
Any AE leading to study drug discontinuation	1 (< 1)	1 (< 1)	2 (< 1)
Any AE leading to study drug interruption	0	1 (< 1)	1 (< 1)
Common AEs (occurring in ≥ 5% of patients)			
Headache	13 (6)	99 (15)	112 (12)
Fatigue	1 (< 1)	80 (12)	81 (9)
Viral upper respiratory tract infection^b^	28 (12)	35 (5)	63 (7)
Nausea	8 (3)	53 (8)	61 (7)
Deaths	0	1 (< 1)^c^	1 (< 1)^c^

*AE* adverse event, *DAA* direct acting antiviral

^a^DAA relatedness determined by study investigator

^b^Adverse events of common cold included per MedDRA version 20.0 that were previously coded as nasopharyngitis in MedDRA version 19.0

^c^Overseas patient died from adenocarcinoma attributed to enlarge peripancreatic nodes and determined to be not related to G/P

Grade ≥ 3 laboratory abnormalities were rare with no Japanese patients experiencing grade ≥ 3 laboratory abnormalities in hemoglobin, alanine aminotransferase (ALT), aspartate aminotransferase (AST), or total bilirubin. Overall, no grade ≥ 3 laboratory abnormalities occurred in hemoglobin or ALT levels. One overseas patient had a Grade 3 AST elevation on Day 41 that returned within normal limits by Day 49. Three overseas (< 1%) patients had Grade 3 elevations in total bilirubin, which were not accompanied by concurrent ALT elevation. There were no events consistent with hepatic decompensation or drug-induced liver injury.

## Discussion

Overall, 8-week G/P treatment was highly efficacious in DAA-naïve patients without cirrhosis and with HCV GT1 or GT2 infection regardless of baseline patient and disease characteristics assessed. Efficacy of 8-week G/P treatment was not impacted by previous predictors of non-response for other DAA regimens including advanced fibrosis, consistent with previous findings from an overseas analysis of G/P [21]. Furthermore, common comorbidities and co-medications in Japan did not impact efficacy of G/P [5]. Overall, this analysis strengthens the evidence from the Japan-only studies, CERTAIN-1 and -2, further supporting 8-week G/P treatment in DAA-naïve patients without cirrhosis and with GT1 or GT2 infection by increasing the sample size in subpopulations of interests, including patients with advanced fibrosis and other common comorbidities. Altogether, in older patient populations particularly those in Japan, United States, and Italy that more commonly present with advanced fibrosis, comorbidities, and co-medication use [5, 6], G/P offers a highly efficacious 8-week treatment option for DAA-naïve patients without cirrhosis and with GT1-2 infection regardless of baseline patient or disease characteristics.

In both Japanese and overseas patients, 8-week G/P treatment was well tolerated with low rates of AEs leading to study drug discontinuation, serious AEs, and laboratory abnormalities including no Grade ≥ 3 ALT elevations. Historically, patients with chronic HCV infection, particularly the elderly, experienced high discontinuations rates with IFN-based regimens [30]; however, IFN-free DAA regimens are safe and well tolerated in all patients, including the elderly, with low rates of AEs leading to study drug discontinuation, serious AEs, and laboratory abnormalities [31, 32]. In both the overseas and Japan-only trials, G/P treatment had infrequent (< 1%) rates of discontinuations due to AEs, DAA-related serious AEs, and grade 3 or higher laboratory abnormalities even in older patients or those treated with G/P for longer durations [22, 23, 33, 34]. In particular, unlike some first-generation protease inhibitor-containing regimens, Grade ≥ 3 ALT elevations were rare (< 1%) and there were no instances consistent with drug-induced liver injury or hepatotoxicity across G/P’s overseas and Japan-only registrational programs [32]. Dedicated clinical trials also previously demonstrated that G/P is safe and well tolerated in a broad range of patients eligible for 8-week G/P treatment including those with CKD stages 4 or 5 and human immunodeficiency virus [25, 35]. In particular, G/P was well tolerated in both overseas and Japanese patients with CKD stages 3, 4, or 5 due to a low rate of AEs leading to discontinuation and no DAA-related serious AEs along with a safety profile consistent with pre-existing comorbidities in the CKD population [35–39]. The higher rates of upper respiratory tract infection in Japanese patients are consistent with high reported rates of this AE (10–35%) observed in Japan-only clinical trials from other DAA regimens [40–42]. Taken together with its efficacy data, 8-week G/P treatment is a safe and efficacious regimen for DAA-naïve patients without cirrhosis from Japan or overseas.

There are limitations to this integrated analysis inherent to its design. There are a low number of patients in some subgroups specifically patients with any NS3 polymorphism, with chronic kidney disease stage 4 or 5, or with a race other than White, Black, or Asian. Patients eligible for 8 week treatment in the real world may be more heterogeneous than this clinical trial population, including patients with underrepresented comorbidities in this analysis. An ongoing Phase 3b clinical trial (EXPEDITION-5) reported that G/P was highly efficacious and well tolerated in patients with HCV GT1-4 infection and CKD stage 3b to 5, including a 96% SVR12 rate and no virologic failures in 84 treatment-naïve or pegIFN/RBV-experienced patients without cirrhosis treated for 8 weeks with G/P [37].

Overall, 8-week G/P treatment is highly efficacious and safe in patients without cirrhosis and with HCV genotype 1 or 2 infection. These data support the current JSH recommendation for 8-week G/P treatment in DAA-naïve patients without cirrhosis and with HCV genotype 1 or 2 infection regardless of baseline patient or viral characteristics [17].

## Electronic supplementary material

Below is the link to the electronic supplementary material.

Supplementary material 1 (DOCX 33 kb)

## Abbreviations

HCV: Hepatitis C virus
GT: Genotype
HCC: Hepatocellular carcinoma
SVR: Sustained virologic response
DAA: Direct acting antiviral
JSH: Japan Society of Hepatology
G/P: Glecaprevir/pibrentasvir
RAS: Resistance-associated substitution
CKD: Chronic kidney disease
IFN: Interferon
RBV: Ribavirin
AST: Aspartate aminotransferase
APRI: Aspartate aminotransferase–platelet ratio index
SVR12: Sustained virologic response at post-treatment week 12
ITT: Intent-to-treat
FIB-4: Fibrosis-4
AE: Adverse event
ALT: Alanine aminotransferase
