# Supplementary material for: Integrated analysis of 8-week glecaprevir/pibrentasvir in Japanese and overseas patients without cirrhosis and with hepatitis C virus genotype 1 or 2 infection
Source: J Gastroenterol. 2019 Mar 13;54(8):752–61. doi: 10.1007/s00535-019-01569-7 (PMC6647445; doi:10.1007/s00535-019-01569-7)
Supplement: Supplementary file 1 — Supplementary material 1 (DOCX 33 kb) [file 535_2019_1569_MOESM1_ESM.docx]

# Online Supporting Information

# Integrated analysis of 8-week glecaprevir/pibrentasvir in Japanese and overseas patients without cirrhosis and with hepatitis C virus genotype 1 or 2 infection

Atsushi Naganuma^1^, Kazuaki Chayama^2^, Kazuo Notsumata^3^, Edward Gane^4^, Graham Foster^5^, David Wyles^6^, Paul Kwo^7^, Eric Crown^8^, Abhi Bhagat^8^, Federico Mensa^8^, Tetsuya Otani^8^, Lois Larsen^8^, Margaret Burroughs^8^, Hiromitsu Kumada^9^

## Table of Contents

HCV RNA Assays…………………………………………………………………………………………………………………………………..2

[Eligibility Criteria 3](#_Toc479596269)

[Inclusion 3](#_Toc479596270)

[Exclusion](#_Toc479596270) 7

Table S1. Characteristics for Each Patient Experiencing Virologic Failure. 10

Table S2. Characteristics of Patients Experiencing Non-Virologic Failure…………………………………………..11

Table S3. Adverse Events in Patients with or without Advanced Liver Fibrosis …...…………………………..12

# HCV RNA Assays

For the patients enrolled in Phase 2 trials as well as those enrolled in the Phase 3 SURVEYOR-II Part 4 study, specimen preparation was done manually with the High Pure System and plasma HCV RNA levels were determined for each sample collected by the central laboratory using the COBAS TaqMan® real-time reverse transcriptase-PCR (RT-PCR) assay v. 2.0 (Roche Molecular Diagnostics, Pleasanton, CA), which has a LLOQ of 25 IU/mL, regardless of genotype. The LLOD is 15.0 IU/mL for genotypes 1 and 5.6 IU/mL for HCV genotype 2. For patients enrolled in Phase 3 trials (excluding those enrolled in SURVEYOR-II Part 4), plasma HCV RNA levels were determined for each sample collected by the central laboratory using the COBAS Ampliprep/TaqMan® real-time reverse transcriptase-PCR (RT-PCR) assay v. 2.0 (Roche Molecular Diagnostics, Pleasanton, CA), which has a lower limit of quantification (LLOQ) and a lower limit of detection (LLOD) of 15 IU/mL, regardless of genotype.

# Eligibility Criteria

## Inclusion

### Male or female, at least 18 years of age at time of Screening.

### If female, subject must be either:

### Practicing one effective method of birth control with male partner(s) from screening to 30 days after stopping study drug

### Postmenopausal for at least 2 years prior to screening

### Or permanently surgically sterile (defined as bilateral tubal ligation, bilateral oophorectomy, or hysterectomy) or has a vasectomized partner(s)

### Females of childbearing potential must have a negative serum pregnancy test result at Screening, and a negative urine pregnancy test at Study Day 1.

### Females of non-childbearing potential (either postmenopausal or permanently surgically sterile) at Screening do not require pregnancy testing.

### Sexually active males must be surgically sterile or have male partners only, or if sexually active with female partner(s) of childbearing potential must agree to practice at least one effective form of birth control

### Screening laboratory result indicating HCV GT1- or GT2-infection; infection with more than one genotype was not permitted.

### Subject has positive anti-HCV Ab and plasma HCV RNA viral load ≥ 1000 IU/mL at Screening Visit.

### Chronic HCV infection defined as one of the following:

### Positive for anti-HCV antibody (Ab) or HCV RNA at least 6 months before Screening; or

### A liver biopsy consistent with chronic HCV infection; or

### Abnormal alanine aminotransferase (ALT) levels for at least 6 months before Screening (only in Phase 3 studies).

### Subject must be HCV DAA treatment-naïve (i.e. subject had not received a single dose of any approved or investigational DAA).

- Prior treatment experience using IFNs with or without RBV was acceptable.
- Previous HCV IFN based treatment had to have been completed ≥2 months prior to Screening

1. Body Mass Index (BMI) is ≥ 18.0 kg/m^2^ at the time of Screening. BMI is calculated as weight measured in kilograms (kg) divided by the square of height measured in meters (m).
2. Subject must be documented as non-cirrhotic defined as meeting one of the following criteria:
   - A liver biopsy within 24 months prior to or during Screening demonstrating the absence of cirrhosis, e.g., a METAVIR, Batts-Ludwig, Knodell, IASL, Scheuer, or Laennec fibrosis score of ≤ 3, Ishak fibrosis score of ≤ 4; or
   - A FibroScan® score of < 12.5 kPa within ≤ 6 months of Screening or during Screening period (FibroScan® must be approved by the local regulatory agency to qualify for entrance criteria); or
     1. Subjects with indeterminate FibroScan® score (12.5≤ score < 14.6), must have a qualifying liver biopsy
   - A Screening FibroTest score and Aspartate Aminotransferase to Platelet Ratio Index (APRI) both indicative of cirrhosis.
     1. Subjects with indeterminate Fibrotest, or conflicting FibroTest and APRI results must have a qualifying liver FibroScan® or biopsy.
   - CERTAIN-1 and -2 ONLY: A Screening Discriminant Score (z) < 0, according to the following formula: z = 0.124 x [gamma-globulin (%)] + 0.001 x [hyaluronate (µg x 1^-1^) -0.075 x [platelet (x 10^4^ cells/mm^3^)] – 0.413 x gender (male, 1; female, 2) – 2.005
     1. Subjects with indeterminate Discriminant Score (score = 0), had a qualifying FibroScan® or liver biopsy
3. Subject must voluntarily sign and date an informed consent form, approved by an Institutional Review Board (IRB)/Independent Ethics Committee (IEC) prior to the initiation of any Screening or study specific procedures.
4. Subjects must be able to understand and adhere to the study visit schedule and all other protocol requirements.

**For ENDURANCE-1 and EXPEDITION-2**

1. Positive test result for anti-Human Immunodeficiency Virus antibody at Screening.
2. Naïve to treatment with any antiretroviral therapy (ART) (and have no plans to initiate ART treatment while participating in this study), or on a stable, qualifying HIV-1 ART regimen for at least 8 weeks prior to Screening. The HIV-1 ART regimen must include at least one of the following ARV agents:
   - For cirrhotic and non-cirrhotic subjects:
     1. Raltegravir (RAL) PO BID
     2. Dolutegravir (DTG) PO QD or PO BID
     3. Rilpivirine (RPV) PO QD
     4. Elvitegravir/cobicistat (EVG/COBI) PO QD
   - For non-cirrhotic subjects, the following regimens are also allowed:
     1. Darunavir (DRV) co-administered with ritonavir (RTV) PO QD
     2. Darunavir/cobicistat (DRV/COBI) PO QD
     3. Lopinavir/ritonavir (LPV/r) PO BID

In addition to the above medications, subjects (both cirrhotic and non-cirrhotic) may take a nucleoside/nucleotide reverse transcriptase inhibitor (N(t)RTI) backbone containing any of the following:

- - Tenofovir disoproxil fumarate (TDF) PO QD
  - Tenofovir alafenamide (TAF) PO QD
  - Abacavir (ABC) PO QD or BID
  - Emtricitabine (FTC) PO QD
  - Lamivudine (3TC) PO QD or BID

Subjects receiving any other HIV-1 ART in addition to those noted above would not be eligible for enrollment in the study.

1. Subjects naïve to ART must have the following:
   - CD4+ count ≥ 500 cells/mm3 (or CD4+ % ≥ 29%) at Screening; and
   - Plasma HIV-1 RNA < 1,000 copies/mL at Screening (by the COBAS® Ampliprep/COBAS® Taqman HIV-1 Test, v 2.0) and at least once during the 12 months prior to Screening (by an approved plasma HIV-1 RNA quantitative assay including but not limited to: COBAS® Ampliprep/COBAS® Taqman HIV-1 Test, v 2.0 or Abbott RealTime HIV-1 assay).
2. Subjects on a stable ART regimen must have the following:
   - CD4+ count ≥ 200 cells/mm3 (or CD4+ % ≥14%) at Screening; and
   - Plasma HIV-1 RNA below LLOQ at Screening (by the COBAS® Ampliprep/COBAS® Taqman HIV-1 Test, v 2.0) and at least once during the 12 months prior to Screening (by an approved plasma HIV-1 RNA quantitative assay including but not limited to: COBAS® Ampliprep/COBAS® Taqman HIV-1 Test, v 2.0 or Abbott RealTime HIV-1 assay).

**For CERTAIN-1 only**

1. Subjects with severe renal impairment, defined as eGFR <30 mL/min/1.73 m^2^, were eligible
   - eGFR calculated using the modification of diet in renal disease (MDRD) method modified for Japanese population including end-stage renal disease on dialysis:

eGFR_J_ = 194 x Serum Creatinine^-1.094^ x Age^-0.287^ x 0.739 [if female]

## Exclusion

1. Female subject who is pregnant, breastfeeding or is considering becoming pregnant during the study; or a male whose partner is pregnant or planning to become pregnant during the study.
2. Recent (within 6 months prior to study drug administration) history of drug or alcohol abuse that could preclude adherence to the protocol in the opinion of the investigator.
3. Subjects on peritoneal dialysis.
4. Positive test result at Screening for hepatitis B surface antigen (HBsAg) or Human Immunodeficiency virus (HIV) Ab.
5. HCV genotype performed during Screening indicating co-infection with more than one HCV genotype.
6. Requirement for and inability to safely discontinue the medications or supplements listed below at least 2 weeks or 10 half-lives (whichever is longer) prior to the first dose of any study drug.
   - Any herbal medicines or supplements (excluding hepatoprotective agents), red yeast rice (monacolin K), St. John’s wort
7. Clinically significant abnormalities or co-morbidities, other than HCV/HIV-1 co-infection, based upon the results of a medical history, physical examination, vital signs, laboratory profile, and a 12-lead electrocardiogram (ECG) that make the subject an unsuitable candidate for this study in the opinion of the investigator, including, but not limited to::

- Uncontrolled diabetes as defined by a glycated hemoglobin (hemoglobin A1C) level > 8.5% during Screening.
- Active or suspected malignancy or history of malignancy (other than basal cell skin cancer or cervical carcinoma in situ) in the past 5 years.
- Uncontrolled cardiac, respiratory, gastrointestinal, hematologic, neurologic, psychiatric, or other medical disease or disorder, which is unrelated to the existing HCV infection..

1. Any cause of liver disease other than chronic HCV-infection, including but not limited to the following:
   - Hemochromatosis.
   - Alpha-1 antitrypsin deficiency.
   - Wilson's disease.
   - Autoimmune hepatitis.
   - Alcoholic liver disease.
   - Steatohepatitis on liver biopsy considered to be the primary cause of the liver disease rather than concomitant/incidental with HCV infection.
2. Screening laboratory analyses showing any of the following abnormal laboratory results:
   - ALT > 10 × ULN
   - AST > 10 × ULN
   - Calculated creatinine clearance (using Cockcroft-Gault method) of < 50 mL/min except in CERTAIN-1
   - Direct bilirubin > ULN
   - Albumin < 3.0 g/dL
   - International normalized ratio (INR) > 1.5 × ULN, unless subject has known hemophilia or is on a stable anticoagulant regimen affecting INR
   - Hemoglobin < 10 g/dL
   - Platelets <90,000 cells per mm^3^ for subjects without cirrhosis
3. History of solid organ transplantation.
4. Receipt of any investigational product within a time period equal to 10 half-lives of the product, if known, or a minimum of 6 weeks (whichever is longer) prior to study drug administration.
5. Any current or past clinical evidence of decompensated liver disease such as ascites noted on physical exam, use of beta-blockers for portal hypertension, hepatic encephalopathy or esophageal variceal bleeding.
6. Consideration by the investigator, for any reason, that the subject is an unsuitable candidate to receive glecaprevir/pibrentasvir.
7. Requirement for chronic use of systemic immunosuppressants including, but not limited to, corticosteroids (prednisone equivalent of > 10 mg/day for > 2 weeks), azathioprine, or monoclonal antibodies (e.g., infliximab).
8. History of severe, life-threatening or other significant sensitivity to any excipients of the study drug.
9. Treatment for an AIDS-associated opportunistic infection (OI) (Appendix E) within 6 months of Screening (only in SURVEYOR-I).
10. Patients who cannot participate in the study per local law.

# Table S1. Characteristics for Each Patient Experiencing Virologic Failure: Prior treatment experience and NS3 and NS5A Polymorphisms^a^

|  |  |  |  | **NS3 Variants^b^** | | **NS5A Variants^b^** | |
| --- | --- | --- | --- | --- | --- | --- | --- |
| **Treatment Duration** | | **HCV Subtype** | **Failure** | **Baseline** | **At Failure** | **Baseline** | **At Failure** |
| **ENDURANCE-1** | |  |  |  |  |  |  |
| 8 weeks | | 1a | Breakthrough | None | A156V | None | Q30R + L31M + H58D |
| **SURVEYOR-II** | |  |  |  |  |  |  |
| 8 weeks | | 2a | Relapse | None | None | L31M | L31M |
| ^a^Detection of polymorphisms was done with next-generation sequencing using a 15% detection threshold. For samples with multiple variants (polymorphisms/substitutions) within a target, if individual variants were detected at ≥90% prevalence , they are considered to be linked and denoted by “+”, whereas if one or more of the variants was detected at <90% prevalence, the variants are separated by a comma  ^b^Amino acid positions included in analysis of patients: 36, 43, 54, 55, 56, 80, 155, 156, and 168 in NS3; 24, 28, 29, 30, 31, 32, 58, 62 (GT1 only), 92, and 93 in NS5A. | | | | | | | |

# Table S2. Characteristics of Patients Experiencing Non-Virologic Failure

| Sex/Age/Race | HCV GT | Reason for non-response | Explanation |
| --- | --- | --- | --- |
| Female/35/White | 1 | Premature G/P discontinuation | Discontinued due to non-compliance with G/P at Day 2 |
| Female/67/White | 1 | AE leading to premature G/P discontinuation and death | Died from adenocarcinoma on day 29 of G/P treatment in a patient with a history of renal insufficiency and hypoechoic lymph nodes; attributed to enlarge peripancreatic nodes and determined to be not related to G/P |
| Male/30/White | 2 | Premature G/P discontinuation | Lost to follow-up after 15 days of G/P treatment |
| Male/48/White | 2 | Premature G/P discontinuation | Withdrew consent; No detectable HCV RNA at last study visit on Day 44 of G/P treatment |
| Male/52/Japanese | 2 | AEs leading to premature G/P discontinuation | Non-serious AEs of nausea and vomiting leading to premature G/P discontinuation at Day 18 |
| Male/50/White | 1 | Missing SVR12 data | No detectable HCV RNA at last visit on Day 71 (15 days after last G/P dose); did not return for SVR12 visit |
| Female/44/Japanese | 1 | Missing SVR12 data | No detectable HCV RNA at last visit (post-treatment week 4); did not return for SVR12 visit |
| Female/43/Japanese | 2 | Missing SVR12 data | No detectable HCV RNA at last visit (end of treatment); did not return for SVR12 visit |
| HCV, Hepatitis C virus; GT, genotype; G/P, glecaprevir/pibrentasvir; AE, adverse event; SVR12, sustained virologic response at 12 weeks post-treatment | | | |

# Table S3. Adverse Events in Patients with or without Advanced Liver Fibrosis

| Event, n (%) | Without Advanced Fibrosis (FIB-4 ≤3.25)  N = 830 | With Advanced Fibrosis (FIB-4 >3.25)  N = 69 |
| --- | --- | --- |
| Any AE, n (%) | 503 (61) | 38 (55) |
| Any DAA-related^a^ AE | 264 (32) | 20 (29) |
| Any serious AE | 12 (1) | 2 (3) |
| Any DAA-related^a^ serious AEs | 0 | 0 |
| Any AE leading to study drug discontinuation | 1 (<1) | 1 (1) |
| Any AE leading to study drug interruption | 1 (<1) | 0 |
| Common AEs (occurring in ≥5% of patients) |  |  |
| Headache | 104 (13) | 8 (12) |
| Fatigue | 78 (9) | 3 (4) |
| Viral upper respiratory tract infection^b^ | 61 (7) | 2 (3) |
| Nausea | 57 (7) | 4 (6) |
| Deaths | 0 | 1 (1)^c^ |
| AE, adverse event; DAA, direct acting antiviral  ^a^DAA relatedness determined by study investigator  ^b^ Adverse events of common cold included per MedDRA version 20.0 that were previously coded as nasopharyngitis in MedDRA version 19.0  ^c^Overseas patient died from adenocarcinoma attributed to enlarge peripancreatic nodes and determined to be not related to G/P | | |
